# Bridging Osteoimmunology and Regenerative Therapy: The Role of MSCs and Extracellular Vesicles

**DOI:** 10.3390/ijms27031155

**Published:** 2026-01-23

**Authors:** Itziar Álvarez-Iglesias, Alice Colombo, Luis Gil-de-Gómez, Daniel García-Sánchez, Alberto González-González, Flor M. Pérez-Campo

**Affiliations:** Departamento de Biología Molecular, Universidad de Cantabria-IDIVAL, 39011 Cantabria, Spain

**Keywords:** mesenchymal stem cells, osteoimmunology, bone regeneration, extracellular vesicles, immunomodulation, cell-free therapy

## Abstract

Bone homeostasis and regeneration depend on tightly regulated interactions between skeletal cells and the immune system within the bone microenvironment. Disruption of this crosstalk by ageing, chronic inflammation, or systemic disease contributes to osteoporosis, inflammatory bone loss, and impaired fracture healing. Osteoimmunology has reframed bone biology as an immune-regulated process, highlighting mesenchymal stem cells (MSCs) as central coordinators of bone-immune communication. Beyond their differentiation capacity, MSCs act primarily through paracrine mechanisms, releasing a secretome composed of soluble factors and extracellular vesicles (EVs) that modulate immune responses, regulate osteoblast and osteoclast activity, promote angiogenesis, and support extracellular matrix remodelling. MSC-derived EVs have emerged as key nanoscale mediators that transfer bioactive cargo to target cells in a context-dependent manner, enabling precise regulation of osteoimmune processes. This review summarises current knowledge on the role of MSCs in osteoimmunology, with a focus on how their secretome and EVs integrate immune modulation with bone regeneration. We discuss the mechanisms underlying MSC-mediated regulation of innate and adaptive immune cells, examine emerging cell-free therapeutic strategies based on secretome and EV delivery, and outline the main challenges that must be addressed to advance these approaches towards clinical application.

## 1. Introduction

Bone is a dynamic and highly specialised tissue whose integrity depends on continuous remodelling and efficient regeneration, thanks to the coordinated activity of osteoblasts, osteoclasts, osteocytes, mesenchymal stem cells (MSCs), and immune cells within a shared microenvironment. Ageing, chronic inflammation, and systemic disease disrupt this crosstalk and shift the balance towards bone resorption, predisposing to osteoporosis, inflammatory bone loss, and impaired fracture healing [1,2].

**Figure 1:**
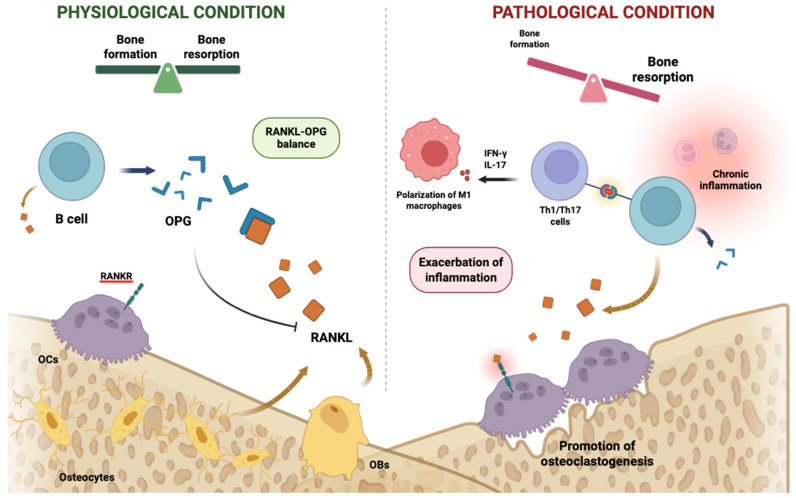
**Immune regulation of bone remodelling under physiological and pathological conditions.** Under physiological conditions (**left**), balanced bone remodelling is maintained through coordinated regulation of osteoblast and osteoclast activity, largely governed by the RANKL-OPG axis. B cells contribute to bone homeostasis by producing osteoprotegerin (OPG), which limits RANKL-mediated osteoclast differentiation. Under pathological conditions characterised by chronic inflammation (**right**), pro-inflammatory Th1/Th17 cells and M1 macrophages produce cytokines such as IFN-γ (context-dependent) and IL-17 (direct), disrupting the RANKL-OPG balance and promoting excessive osteoclastogenesis. This inflammatory shift favours bone resorption over formation, contributing to inflammatory bone loss. Exacerbation of inflammation denotes the sustained activation of pro-inflammatory cytokine circuits that disrupt immune–skeletal coupling and impair regulatory control of the RANKL-OPG axis. While IL-17 directly promotes osteoclastogenesis, IFN-γ exerts predominantly inhibitory effects on osteoclast differentiation by interfering with RANKL signalling; its contribution to bone loss in pathological settings is indirect and context-dependent, occurring primarily through sustained antigen-driven inflammation. Created in BioRender. Pérez-Campo, F. (2026) (https://BioRender.com/ciyhh4v) accessed on 15 January 2026.

The field of osteoimmunology has reframed bone biology as an immune-regulated process rather than a purely skeletal phenomenon. Initially described as a dialogue between T cells and osteoclasts, bone remodelling is now understood as a complex network in which innate and adaptive immune cells regulate bone turnover through cytokines, chemokines, and direct cell-to-cell interactions [1,2]. Pro-inflammatory mediators such as TNF-α, IL-1β, and IL-17 promote osteoclastogenesis and inhibit osteoblast function, thereby driving bone loss. In contrast, Th1-derived IFN-γ exerts predominantly inhibitory effects on osteoclast differentiation by interfering with RANKL signalling through mechanisms such as TRAF6 degradation and suppression of JNK/NF-κB pathways, although indirect pro-resorptive effects have been reported in vivo under conditions of sustained antigen-driven T cell activation [3]. Anti-inflammatory cytokines such as IL-10 support inflammation resolution, while TGF-β exerts stage-dependent effects on bone remodelling, promoting early osteoblast proliferation and chondrogenesis but inhibiting terminal osteoblast differentiation and mineralisation at later stages and variably influencing osteoclast activity depending on the inflammatory context [4,5,6,7,8]. Therefore, both the type and the timing of the immune response play a central role in determining how bone injuries heal and how chronic skeletal diseases progress (Figure 1). When this tightly regulated balance among pro-inflammatory, anti-inflammatory, and stage-dependent signals fails, immune responses can shift from a controlled, reparative process to a persistent pathological state.

In pathological settings, “exacerbation of inflammation” refers to the persistence and amplification of pro-inflammatory cytokine networks, including TNF-α, IL-1β, and IL-17, which override regulatory feedback mechanisms controlling bone remodelling. Sustained inflammatory signalling disrupts the *RANKL-OPG* axis, skews osteoblast-osteoclast coupling towards resorption, and prevents the resolution phase required for effective bone regeneration.

Within this context, MSCs occupy a central position at the interface between bone and immunity. According to the minimal criteria established by the International Society for Cellular Therapy, MSCs are defined by their adherence to plastic under standard culture conditions, expression of the surface markers *CD105*, *CD73*, and *CD90*, lack of expression of haematopoietic and endothelial markers such as *CD45*, *CD34*, *CD14*, or *CD11b*, *CD79a* or *CD19*, and *HLA-DR*, and their capacity to differentiate in vitro into osteoblasts, chondrocytes, and adipocytes [9]. Although originally proposed to harmonise MSC characterisation across laboratories, these criteria remain essential for interpreting functional studies, as deviations from this definition may contribute to phenotypic and functional heterogeneity.

Classically defined by their capacity to differentiate into osteoblasts, chondrocytes, and adipocytes, MSCs are now considered as potent immunoregulatory cells able to sense inflammatory signals and remodel the local microenvironment. They secrete a mix of cytokines, chemokines, growth factors, enzymes, and extracellular vesicles (EVs), overall known as the “secretome”, that modulate the behaviour of immune and stromal cells [10,11]. In fact, rather than long-term engraftment, many of the beneficial effects of MSCs in preclinical models of tissue repair are now attributed to these paracrine mechanisms [12,13]. Among the components of the secretome, extracellular vesicles (EVs) have emerged as particularly relevant mediators of osteoimmunological communication. These lipid bilayer vesicles (mainly exosomes and microvesicles) carry a bioactive cargo including proteins, lipids, and non-coding RNAs that can reprogramme recipient cells in a context-dependent manner [14,15]. MSC-derived EVs influence osteoblast and osteoclast differentiation, modulate macrophage polarisation, and affect T cell responses, thus integrating osteogenic, angiogenic, and immunomodulatory signals within the bone niche [1,5,15,16]. Conversely, EVs released by immune cells can act on MSCs and bone cells, adding an additional layer of regulation to the bone-immune axis [14].

These findings have prompted the development of cell-free strategies based on MSCs’ secretome or isolated EVs as alternatives to cell transplantation. Such approaches could reduce immunogenicity and tumourigenic risk while improving standardisation, storage, and large-scale manufacturing [17,18]. Preclinical data indicate that MSC-derived EVs can enhance fracture repair, attenuate inflammation-associated bone loss, and promote vascularised bone regeneration [1,5,15,16]. However, translation to the clinic still faces substantial challenges, including heterogeneity in EV preparations, incomplete mechanistic understanding, and the need for targeted delivery to skeletal sites.

In this review, we summarise current knowledge on the osteoimmunological roles of MSCs and their EVs, focusing on how they integrate immune regulation with bone regeneration. We then discuss emerging therapeutic strategies that exploit MSCs’ secretome and EVs to modulate immune responses while promoting regeneration and outline the key obstacles that must be overcome to move these approaches towards clinical application.

## 2. Mesenchymal Stem Cells as Coordinators of Bone-Immune Communication

MSCs are increasingly recognised as central regulatory elements within the bone marrow niche, where they integrate immunological signals with the skeletal remodelling processes. Their biological relevance extends beyond their classical definition as multipotent progenitors, as MSCs can sense inflammatory signals and translate them into coordinated responses that influence both bone formation and immune activity. This dual capacity, that is, combining differentiation potential with broad immunomodulatory functions, makes them key determinants of bone homeostasis under physiological conditions, as well as during inflammation and disease [11,19]. Through a combination of direct cell-to-cell interactions and paracrine signalling, MSCs regulate the behaviour of osteoblasts, osteoclasts, macrophages, dendritic cells, and T and B lymphocytes, thus contributing to the fine-tuned balance between bone formation and resorption.

### 2.1. Phenotypic and Functional Features of MSCs in the Bone Marrow Niche

Bone marrow MSCs are primarily located in perivascular and endosteal regions, where they play essential roles in supporting hematopoiesis and maintaining stromal organisation. Within these niches, MSCs interact closely with endothelial cells, hematopoietic stem cells, and immune populations, contributing to the structural and functional integrity of the bone marrow microenvironment. Their low expression of HLA class II molecules and co-stimulatory receptors such as *CD40* confers a relatively low immunogenic profile, enabling MSCs to modulate immune cell activation without eliciting strong immune responses themselves [11].

In addition to their capacity to differentiate into osteoblasts and chondrocytes, MSCs produce cytokines, chemokines, extracellular matrix proteins, and growth factors that coordinate the responses from local tissues. Importantly, MSC dysfunction has been implicated in inflammatory and degenerative bone diseases, where chronic immune activation disrupts the microenvironment and negatively affects osteogenic differentiation. This interaction between stromal dysregulation and immune imbalance is now considered a critical driver of osteoporosis, rheumatoid arthritis, and other osteoimmune pathologies [1,4].

### 2.2. MSC Crosstalk with Innate Immune Cells

Macrophages are central in establishing the inflammatory environment in bone, and their interaction with MSCs has a major impact on bone healing. MSCs influence macrophage behaviour primarily through the release of anti-inflammatory mediators such as interleukin-10 (IL-10), prostaglandin E2, and indoleamine 2,3-dioxygenase (IDO). Through these signals, MSCs promote a shift from pro-inflammatory M1 macrophages towards a reparative M2 phenotype [10,11,20].

M2 macrophages support bone regeneration by enhancing osteoblast differentiation, promoting extracellular matrix deposition, and facilitating angiogenesis. This creates a pro-regenerative microenvironment in which immune resolution and tissue repair progress in parallel. In contrast, sustained activation of M1 macrophages leads to elevated levels of TNF-α, IL-1β, and IL-6, which stimulates osteoclastogenesis and inhibits osteoblast maturation. Such conditions are characteristic of chronic inflammatory bone loss and contribute to impaired healing [1,21].

MSC-mediated reprogramming of macrophages is thus a central mechanism coupling immune resolution with bone repair.

### 2.3. MSC Modulation of Adaptive Immunity

#### 2.3.1. Regulation of T Cell Subsets

MSCs exert context-dependent immunomodulatory effects on T cell activation and proliferation, mediated by a combination of soluble factors including IL-10, TGF-β, hepatocyte growth factor, and indoleamine 2,3-dioxygenase (IDO). Depending on environmental cues and inflammatory priming, MSCs can adopt either a pro-inflammatory (MSC1) or an immunosuppressive (MSC2) phenotype, thereby exerting divergent effects on T cell responses [10,11,22]. Through these mechanisms, MSCs limit excessive T cell activation and promote immune tolerance within the bone marrow niche. MSCs promote the expansion of regulatory T cells (Tregs), which are abundant in the bone marrow, and play essential roles in maintaining hematopoietic and skeletal homeostasis. MSCs support Treg expansion and stability, partly through CXCL12 signalling, highlighting the reciprocal nature of stromal–immune communication [4,19].

**Figure 2:**
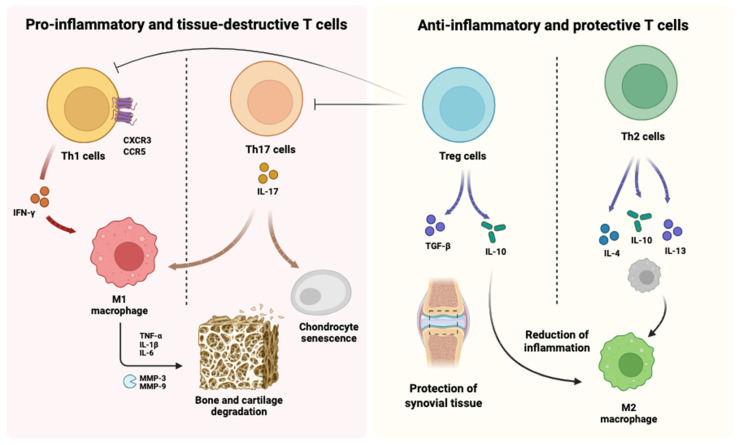
**Differential roles of T cell subsets in osteoimmune regulation.** Pro-inflammatory T cell subsets contribute to tissue damage through distinct and context-dependent mechanisms. Th17 cells promote osteoclastogenesis and inflammatory tissue damage primarily via IL-17–mediated activation of osteoclast precursors and macrophages. In contrast, Th1-derived IFN-γ generally exerts inhibitory effects on osteoclastogenesis by targeting precursor differentiation and fusion, although indirect pro-resorptive effects may occur under conditions of sustained immune activation. Conversely, anti-inflammatory and protective T cell populations, including regulatory T cells (Tregs) and Th2 cells, secrete cytokines such as IL-10, TGF-β, IL-4, and IL-13, favouring immune resolution, M2 macrophage polarisation, and protection of joint and bone tissues. The balance between these opposing T cell programmes critically determines inflammatory versus regenerative outcomes within the skeletal microenvironment. Created in BioRender. Pérez-Campo, F. (2026) (https://BioRender.com/my34id4). accessed on 15 January 2026.

Under inflammatory conditions, an imbalance between T helper 17 (Th17) cells and Tregs drives pathological bone resorption: Th17-derived IL-17 enhances osteoclastogenesis and synovial inflammation, whereas Tregs attenuate these effects through IL-10 and TGF-β release [7,23,24]. By reinforcing Treg activity and limiting Th17 responses, MSCs play a critical role in protecting bone from inflammation-induced degradation [7,23,24] (Figure 2).

#### 2.3.2. Interactions with B Cells

B lymphocytes contribute to bone homeostasis through the production of osteoprotegerin (OPG) and regulation of the RANKL-RANK signalling axis. MSCs modulate B cell behaviour by downregulating chemotactic receptors such as *CXCR4*, *CXCR5*, and *CXCR7*, and by suppressing *STAT3* activation, thereby dampening B cell activation and immunoglobulin production [25]. In inflammatory settings, B cells can become a significant source of RANKL accelerating osteoclastogenesis and contributing to subchondral bone loss in diseases such as osteoarthritis and rheumatoid arthritis [26,27]. By reducing B cell activation and limiting *RANKL* expression, MSCs buffer pathological bone resorption driven by adaptive immune dysregulation.

### 2.4. MSCs as Paracrine Regulators of Bone Remodelling

Although differentiation of MSCs into osteoblasts is fundamental for bone formation, growing evidence indicates that their paracrine activity plays an equally important, and often dominant, role in skeletal repair. MSCs secrete growth factors such as bone morphogenetic proteins (BMPs), vascular endothelial growth factor (VEGF), and TGF-β, together with cytokines and extracellular matrix-modifying enzymes that stimulate osteogenesis, angiogenesis, and stromal regeneration [10]. Beyond skeletal-specific models, the relevance of MSCs-derived paracrine signalling has also been demonstrated in a variety of regenerative and biomaterial-based platforms, where secretome-mediated effects contribute to matrix remodelling, angiogenesis, and tissue repair. Although these approaches extend beyond bone-focused applications, they provide complementary evidence supporting the central role of MSC paracrine activity as a driver of regeneration across different tissue contexts [28,29].

In parallel, the MSC secretome attenuates inflammatory cascades within the bone microenvironment, reducing the inhibitory effects of chronic cytokine exposure on osteoblast maturation and limiting osteoclast activation. Through this integrated paracrine network, MSCs coordinate immune resolution with matrix deposition and vascular support, ensuring that bone regeneration proceeds in a controlled and efficient manner.

Disruption of this paracrine regulation, as occurs during ageing or chronic inflammation, compromises the regenerative capacity of MSCs and contributes to skeletal fragility. These observations reinforce the concept that MSCs act as central “conductors” of the bone remodelling process, integrating multiple biological signals to maintain tissue integrity [1,30].

### 2.5. Implications for Osteoimmune Pathophysiology

The interplay between MSCs and immune cells has direct relevance to disorders such as osteoporosis, osteoarthritis, rheumatoid arthritis, and delayed fracture healing. Excessive production of inflammatory cytokines (TNF-α, IL-1β, IL-17) disrupts MSC osteogenic differentiation while increasing *RANKL* expression, thereby shifting remodelling toward resorption [6,27]. Conversely, enhancing MSC-driven immunoregulation can reverse these pathological processes by re-establishing M2 macrophage dominance, restoring Treg-Th17 balance, and promoting angiogenic–osteogenic coupling.

These findings highlight MSCs as a foundational element in osteoimmunology and justify the growing interest in leveraging MSC-derived secretome and extracellular vesicles as cell-free strategies for bone regeneration.

## 3. Secretome and Extracellular Vesicles as Mediators of MSC-Driven Osteoimmune Regulation

Rather than relying on long-term engraftment, MSCs exert their regulatory effects within the bone microenvironment mainly through paracrine signalling. This section focuses on how MSCs-derived soluble factors and EVs act as key mediators of osteoimmune communication, integrating immune modulation with skeletal remodelling. By influencing inflammatory resolution, osteogenic differentiation, angiogenesis, and extracellular matrix dynamics, these paracrine signals provide a mechanistic link between immune regulation and tissue regeneration [10,11].

### 3.1. Soluble Components of the MSC Secretome in Immune and Skeletal Regulation

#### 3.1.1. Anti-Inflammatory Cytokines and Immunoregulatory Factors

A defining characteristic of MSCs’ secretome is its immunoregulatory profile. MSCs-derived cytokines and mediators such as IL-10, TGF-β, PGE2, indoleamine 2,3-dioxygenase (IDO), and hepatocyte growth factor (HGF) play essential roles in avoiding excessive immune activation. These factors suppress pro-inflammatory Th1 and Th17 responses, inhibit monocyte differentiation into dendritic cells, and limit natural killer cell proliferation, thereby promoting a shift towards immune tolerance [10,25,31]. At the mechanistic level, increasing evidence indicates that the immunomodulatory effects attributed to MSCs-derived paracrine factors converge on intracellular signalling pathways that regulate inflammation, oxidative stress, and cell survival. Experimental studies in inflammatory and regenerative models have shown that modulation of pathways such as PI3K/AKT, redox-sensitive signalling and apoptosis-related cascades can profoundly influence immune cell activation states, cytokine production and tissue repair outcomes. Moreover, advances in nano-encapsulation and targeted delivery strategies have demonstrated that paracrine mediators can exert context-dependent immunoregulatory effects by shaping local microenvironments, including the regulation of angiogenic and chemotactic cues such as VEGF and CCL2. Although these studies are not limited to skeletal tissues, they provide relevant mechanistic frameworks that support the concept that MSCs-derived secretome components act through conserved signalling nodes to coordinate immune suppression, inflammation resolution and regenerative responses [32,33,34].

Within the bone marrow niche, IL-10 and TGF-β are particularly important for maintaining immune balance. These cytokines support the expansion and stabilisation of regulatory T cells (Tregs), which are abundant in bone marrow and protect against inflammation-induced bone degradation [4]. By reinforcing Treg-mediated immune control, MSC-derived factors help prevent the sustained inflammatory signalling that would otherwise impair osteoblast’s function and favour osteoclast activation [4].

Importantly, the immunosuppressive activity of the MSCs’ secretome is context-dependent. In inflammatory environments, exposure to cytokines such as IFN-γ or TNF-α can enhance the expression of IDO and PGE2, amplifying the ability of MSCs to restrain immune activation. This adaptive behaviour allows MSCs to fine-tune immune responses according to local needs, rather than inducing broad immunosuppression.

#### 3.1.2. Pro-Regenerative Growth Factors and Matrix Remodelling Enzymes

The MSCs’ secretome contains various growth factors that directly support skeletal regeneration. Bone morphogenetic proteins (BMPs), vascular endothelial growth factor (VEGF), TGF-β isoforms, and angiogenic mediators support osteoblast maturation, endothelial recruitment, and mineralised matrix deposition. These signals are essential for the formation of mineralised matrix and for sustaining the vascular network required to support new bone tissue.

Matrix metalloproteinases (MMPs) released by MSCs contribute to extracellular matrix remodelling by degrading matrix components and releasing growth factors sequestered in their latent forms, such as TGF-β and VEGF. This controlled remodelling facilitates cell migration, angiogenic sprouting, and the integration of newly formed tissue during repair [35].

Through the combined action of growth factors and matrix-remodelling enzymes, soluble components of the MSC secretome link immune resolution with the initiation and progression of osteogenesis and neovascularisation. These processes are tightly coordinated, ensuring that bone formation occurs in an environment permissive for regeneration rather than chronic inflammation.

### 3.2. Modulation of Immune Cell Phenotypes by the MSC Secretome

#### 3.2.1. Macrophage Reprogramming and Inflammation Resolution

One of the most robust immunomodulatory actions of the MSC secretome is the redirection of macrophages from pro-inflammatory M1 states toward a reparative M2 phenotype. IL-10, PGE2, and IDO promote the transition of macrophages from a pro-inflammatory M1 state towards an anti-inflammatory M2 state. This shift is accompanied by a reduction in M1-associated cytokines such as TNF-α, IL-1β, and IL-6, and by increased expression of factors that support angiogenesis and tissue repair [25,36,37].

M2 macrophages play a critical role in bone regeneration by supporting osteoblast differentiation and coordinating vascular responses. In contrast, sustained M1 activation is a hallmark of inflammatory bone loss and contributes to impaired healing by enhancing osteoclastogenesis and suppressing osteoblast maturation. By actively shaping macrophage behaviour, the MSCs’ secretome links immune resolution to the restoration of a regenerative bone microenvironment [1].

**Figure 3:**
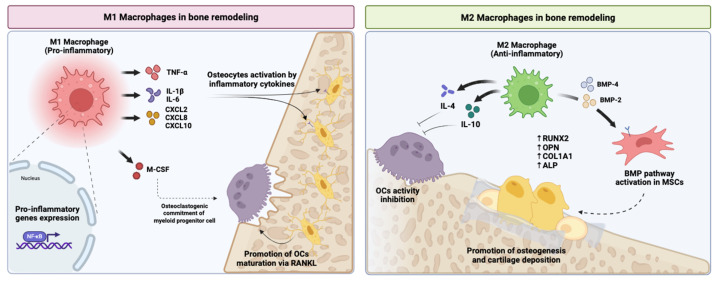
**Macrophage polarisation as a determinant of bone remodelling outcomes.** Pro-inflammatory M1 macrophages secrete cytokines and chemokines such as TNF-α, IL-1β, IL-6, CXCL2, CXCL8, and CXCL10, activating inflammatory gene expression programmes and promoting osteoclast differentiation and maturation via RANKL signalling. In contrast, anti-inflammatory M2 macrophages release cytokines including IL-4 and IL-10, inhibit osteoclast activity, and support osteogenesis by enhancing BMP signalling in MSCs, leading to increased expression of osteogenic markers such as *RUNX2*, *OPN*, *COL1A1*, and *ALP*. The dynamic balance between M1 and M2 macrophages links immune resolution to bone formation and cartilage deposition. The arrow labelled “osteoclastogenic commitment of myeloid progenitor cell” refers to the biased differentiation of myeloid progenitors toward the osteoclast lineage under M1-skewed inflammatory conditions. Created in BioRender. Pérez-Campo, F. (2026) (https://BioRender.com/uezxl7d). accessed on 15 January 2026).

This macrophage-directed effect highlights the indirect yet powerful influence of MSC-derived factors on skeletal repair, acting upstream of osteoblast and osteoclast responses by first normalising the inflammatory context (Figure 3).

#### 3.2.2. Regulation of T Cell and B Cell Responses

Beyond innate immunity, MSC-secreted factors exert significant control over adaptative immune responses. MSCs-derived mediators inhibit T cell proliferation in a dose-dependent manner and modulate T cell differentiation by suppressing TH17 effector activity, while reinforcing Treg-mediated immune control [25,38,39]. This shift is particularly relevant in osteoimmune diseases, where excessive Th17 responses promote osteoclastogenesis and sustain chronic inflammation.

MSCs also regulate B cell behaviour through both chemokine-mediated and intracellular signalling mechanisms. By downregulating *CXCR4*, *CXCR5*, and *CXCR7* signalling and inhibiting STAT3 activation, MSC-derived factors reduce B cell activation, plasma cell differentiation, and antibody production [40,41]. In inflammatory settings, where B cells can become a major source of RANKL and drive osteoclast formation, this regulatory effect contributes to the preservation of bone integrity [26,27].

Through coordinated modulation of T cell and B cell responses, the MSCs’ secretome attenuates immune-driven osteoclastogenesis and reinforces mechanisms that favour bone stability under inflammatory conditions.

### 3.3. Extracellular Vesicles: Nanoscale Mediators of Osteoimmune Crosstalk

#### 3.3.1. Biogenesis and Functional Repertoire of MSC-Derived EVs

Extracellular vesicles derived from MSCs, including exosomes and microvesicles, carry a cargo of proteins, lipids, mRNAs, and microRNAs that reflect both their cell of origin and the physiological state of the producing MSCs [14,42]. As such, EVs act as structured packages of biological information rather than passive by-products of cell metabolism. MSCs-derived EVs deliver this cargo to osteoblasts, osteoclast precursors, endothelial cells, macrophages, and T cells. Through this targeted delivery, EVs precisely regulate skeletal and immune processes within the bone marrow niche [15,16].

#### 3.3.2. Osteogenic Effects Mediated by EV Cargo

Numerous studies demonstrate that MSC-derived EVs enhance osteoblast differentiation and mineralisation through the transfer of microRNAs that target inhibitors of osteogenesis. miRNAs that are part of the EVs’ cargo, such as miR-21-5p, miR-196a, miR-148a-3p, and miR-140-3p, have been shown to activate *RUNX2* and BMP signalling and promote the deposition of extracellular matrix in vitro and in vivo [43,44,45,46,47].

The osteogenic potential of MSCs-derived EVs can be further enhanced by preconditioning these cells prior to vesicle isolation. Hypoxic culture, exposure to biomaterials, or osteogenic induction enrich EV cargo with pro-osteogenic miRNAs such as miR-210-3p, miR-27a-3p, and miR-126, producing vesicles with increased reparative capacity [48,49,50]. These findings underscore the plasticity of EV composition and its dependence on MSC environmental cues.

#### 3.3.3. Immune Modulation Through EV-Mediated Signalling

**Figure 4:**
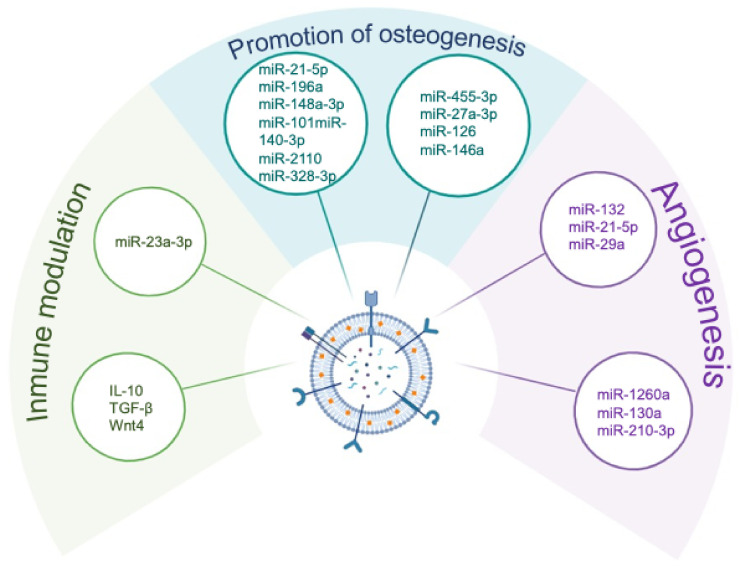
**Molecular cargo of mesenchymal stem cell–derived extracellular vesicles involved in osteoimmune regulation.** Schematic representation of key bioactive mediators identified in extracellular vesicles (EVs) released by mesenchymal stem cells (MSCs) and their functional contribution to osteoimmune crosstalk. MSC-derived EVs transport regulatory microRNAs and soluble factors that collectively modulate immune responses, promote osteogenic differentiation, and support angiogenesis within the bone microenvironment. Immunomodulatory cargo includes microRNAs and cytokine-related mediators that favour inflammation resolution, whereas osteogenesis-associated microRNAs contribute to osteoblast commitment and bone matrix formation. In parallel, EV-associated angiogenic microRNAs support vascularisation, which is tightly coupled to bone regeneration. Together, this multifunctional EV cargo underlies the capacity of MSC secretome to integrate immunomodulatory, osteogenic, and angiogenic signals in skeletal tissues.

MSC-derived EVs can suppress inflammatory signalling by delivering microRNAs that block inflammatory signalling pathways. Several EV-associated microRNAs inhibit NF-κB activation, reduce inflammasome activity, and promote M2 macrophage polarisation. For example, miR-23a-3p supports macrophage reprogramming toward anti-inflammatory phenotypes, while miR-21-5p modulates TLR4/NF-κB signalling [51,52]. In addition, EVs have also been shown to inhibit ubiquitination pathways involved in NF-κB and mTOR activation, further reducing inflammatory cascades in preclinical models [53]. Through these mechanisms, EVs act as carriers that integrate osteogenic and immunoregulatory signals, reinforcing the concept of vesicle-mediated communication as a central axis of osteoimmune regulation (Figure 4). In addition to regulatory microRNAs, MSC-derived extracellular vesicles and the broader MSCs secretome encompass immunomodulatory proteins that contribute to osteoimmune regulation. These include cytokines such as IL-10 and TGF-β, which play central roles in inflammation resolution and immune tolerance, as well as signalling molecules such as Wnt4, which has been implicated in macrophage polarisation and immune–stromal crosstalk in regenerative microenvironments. While these factors are classically described as soluble mediators, accumulating evidence indicates that selected cytokines and Wnt ligands can also be associated with extracellular vesicles or act in close functional coordination with EV-mediated signalling (Figure 4).

### 3.4. Angiogenic and Vascular-Regenerative Roles of MSC-Derived EVs

Successful bone regeneration requires adequate vascular support, and MSC-derived EVs contribute significantly to angiogenic processes. These vesicles promote endothelial proliferation, migration, and tube formation through the delivery of microRNAs such as miR-132, miR-29a, miR-21-5p, and miR-130a. By targeting regulators including *RASA1*, *VASH1*, *HDAC7*, and components of the PTEN/AKT pathway, EVs activate angiogenic signalling cascades that support neovascularisation [51,54,55,56].

Embedding EVs within biomaterial scaffolds or hydrogels further enhances their therapeutic potential by improving local retention and sustained release. In vivo studies show that such delivery systems amplify vascularised bone regeneration, demonstrating that EV-mediated angiogenesis is a key contributor to effective skeletal repair [57,58].

Despite these well-documented regenerative effects, increasing evidence indicates that the immunomodulatory activity of MSC-derived secretome and EVs is highly context-dependent, highlighting the need for a critical assessment of their potential limitations.

### 3.5. Context-Dependent Effects and Potential Limitations of MSC-Derived Secretome and Extracellular Vesicles

While a growing body of evidence supports the regenerative and immunomodulatory properties of MSCs-derived secretomes, it is now clear that these effects are highly context-dependent rather than universally beneficial. Immune regulation is an intrinsically dynamic process, and signalling pathways that promote inflammation resolution, angiogenesis, or tissue repair in acute or well-controlled settings may exert neutral or even adverse effects when sustained, excessive, or activated in inappropriate pathological contexts [59,60]. Accordingly, the biological activity of the MSC secretome should be interpreted within the specific temporal, cellular, and microenvironmental conditions in which it operates.

A clear illustration of this duality is macrophage polarisation towards the M2-like phenotype, which is frequently associated with inflammation resolution, extracellular matrix remodelling, and regenerative outcomes. While M2 macrophages support tissue repair during acute injury, prolonged or dysregulated M2 polarisation has been linked to pathological fibrosis, defective tissue remodelling, and tumour progression in chronic inflammatory and oncological settings [61,62]. These observations suggest that excessive or sustained secretome-mediated skewing of macrophage phenotypes may compromise tissue homeostasis or favour wrong repair responses, depending on the disease context.

Similarly, several immunomodulatory mediators enriched in MSC-derived soluble factors and EVs, including TGF-β, IL-10, and pro-angiogenic factors, display pleiotropic and stage-dependent effects. Although these signals are essential for immune resolution and early regenerative processes, their prolonged or uncontrolled activation has been associated with fibrotic responses, altered immune surveillance, and aberrant stromal activation [8,60]. This dual behaviour highlights the importance of tightly regulating the intensity, duration, and localisation of secretome-mediated signalling when considering therapeutic applications.

Beyond macrophages, MSCs themselves exhibit marked functional plasticity in response to inflammatory cues, adopting either pro-inflammatory (MSC1) or immunosuppressive (MSC2) phenotypes. This polarisation directly influences both the composition and the biological activity of the secretome and EV cargo, potentially leading to divergent immunological outcomes across experimental and clinical settings [22]. Such plasticity provides a plausible explanation for the heterogeneous and sometimes contradictory results reported in immunomodulation studies and reinforces the need for a context-aware interpretation of MSCs-based therapeutic strategies.

## 4. Translational Development of the MSC Secretome and Extracellular Vesicles for Bone Repair

### 4.1. Rationale for Advancing Cell-Free Regenerative Strategies

The growing recognition that MSCs exert most of their therapeutic actions through paracrine and vesicle-mediated mechanisms has redirected the field of regenerative medicine toward cell-free approaches. Numerous preclinical studies demonstrate that, following transplantation, MSCs exhibit only limited survival and engraftment within damaged tissues, yet their beneficial effects persist well beyond their physical presence. This has shifted the conceptual framework from relying on MSC differentiation to harnessing the wide array of bioactive mediators they release, including cytokines, chemokines, growth factors, matrix-remodelling enzymes, and EVs. These paracrine factors act on multiple cellular targets, such as osteoblasts, osteoclasts, osteocytes, macrophages, and lymphocytes, to restore the balance between bone formation, resorption, vascularisation, and inflammatory resolution [10,11].

Cell-free MSC-based therapies provide key advantages over cellular transplantation. They mitigate risks associated with cell viability and uncontrolled differentiation, reduce immunogenicity and tumourigenic potential, and offer improved compatibility with GMP-compliant manufacturing pipelines. Secretome- and EV-based products can be stored, standardised, and distributed as off-the-shelf therapeutics, enabling broader clinical implementation and facilitating reproducibility. Their enhanced stability compared with live cells opens possibilities for long-term storage and repeated administration without compromising biological potency [18,63,64].

A substantial body of evidence supports their relevance for bone pathologies characterised by inflammatory deregulation, including osteoporosis [25,65] and osteoarthritis [66,67]. In osteoimmune diseases, EVs derived from MSCs downregulate key inflammatory mediators, suppress osteoclast activity, and restore osteoblast function, thereby limiting bone loss associated with chronic cytokine exposure [1]. Furthermore, in fracture models, MSC-EVs enhance callus formation, promote endothelial recruitment, accelerate mineral deposition, and improve the mechanical properties of the newly formed bone [5,15,16]. Together, these findings emphasise that recapitulating the MSC secretory profile is a powerful strategy capable of engaging both immune and skeletal compartments, supporting the transition of cell-free products into translational pipelines for orthopaedic applications.

### 4.2. Strategies for Enhancing the Therapeutic Potency of MSC Secretome and EVs

#### 4.2.1. Preconditioning and Environmental Modulation of MSCs

Enhancing the therapeutic potential of the MSC secretome begins with modulating the conditions under which MSCs are cultured. Hypoxic preconditioning is among the most extensively studied approaches, reflecting the physiological conditions of the bone marrow niche. Under low oxygen tension, stabilisation of HIF-1α leads to the release of EVs enriched in angiogenic and osteogenic microRNAs, including miR-210-3p and miR-126. These EVs promote endothelial sprouting, enhance cell survival, and stimulate osteoblast differentiation, significantly improving bone regeneration in vivo [48,68]. Hypoxic preconditioning therefore acts as a potent driver of EV-mediated signalling, aligning the vesicular cargo with the metabolic requirements of early tissue repair.

Biomaterial-based modulation offers another powerful means of conditioning MSCs. Exposure to lithium-containing or strontium-substituted ceramics induces intracellular pathways that result in the enrichment of EV cargo with osteoinductive and angiogenic microRNAs such as miR-130a and miR-146a. These changes have been shown to accelerate mineralisation and increase microvascular density in preclinical bone defect models [54,69]. Mechanical stimulation–such as microvibration or magnetic cues—further modifies the EV secretion profile, increasing vesicle yield and enhancing the representation of microRNAs involved in osteogenic specification [58]. Collectively, these strategies highlight the dynamic adaptability of MSC paracrine activity and demonstrate how environmental cues can be intentionally harnessed to engineer more potent regenerative products.

Inducing MSCs toward the osteogenic lineage prior to EV collection also produces vesicles particularly suited for bone repair. Osteogenically preconditioned EVs contain transcripts and microRNAs that activate *RUNX2*, BMP2, and other key regulators of osteoblast differentiation, thereby accelerating mineralised matrix deposition both in vitro and in vivo [49]. Each modality—hypoxic, biomaterial-driven, mechanical, or instructive biochemical stimulation—offers distinct advantages, yet all converge on a shared outcome: the generation of a secretome with markedly enhanced regenerative capacity.

#### 4.2.2. Genetic Engineering Approaches to Enhance EV Content

Genetic modification of MSCs offers a more direct and precise method for tailoring EV cargo. Overexpression of microRNAs such as miR-21-5p or miR-29a results in EVs with potent dual osteogenic and angiogenic activity by targeting pathways including Akt, VEGF, and BMP signalling [44,55]. These engineered EVs can induce robust osteoblast differentiation while concurrently promoting neovascularisation, two processes essential for stable bone regeneration.

Similarly, overexpression of anti-resorptive microRNAs such as miR-6924-5p produces EVs capable of attenuating osteoclastogenesis by interfering with RANKL-mediated activation of osteoclast precursors [70]. Beyond RNA cargo, EVs can be engineered to carry therapeutic proteins, facilitated by sequence motifs that enhance protein loading into the vesicular lumen [71,72]. This strategy expands the potential of EVs beyond gene regulation to include intracellular delivery of active enzymes or signalling regulators [73]. Collectively, genetically engineered MSC-EVs represent a new generation of rationally designed biologics that combine targeted immune modulation with enhanced bone-forming potential.

### 4.3. Biomaterial-Based Delivery Systems for the Secretome and EVs

#### 4.3.1. Hydrogels and Structural Scaffolds

Achieving sustained and localised delivery of EVs to bone defects is a central challenge for clinical translation. Hydrogels have emerged as effective carriers due to their biocompatibility, high water content, and capacity to protect EV integrity while permitting controlled release. Thermosensitive hydrogels encapsulating EVs enriched in miR-21 have demonstrated superior vascularised bone formation compared with bolus injection, as the matrix prolongs EV presence at the defect site and supports early vascular infiltration [58]. Collagen-based hydrogels and biodegradable polymer matrices create three-dimensional environments conducive to cellular migration and osteogenic commitment, while maintaining vesicle bioactivity [57].

Composite scaffolds combining polymers and bioactive ceramics provide structural support and simultaneously serve as reservoirs for sustained EV release. When functionalised with MSCs-derived EVs, these scaffolds promote accelerated mineral deposition and enhance the biomechanical properties of regenerated bone [74,75]. Their architectural similarity to trabecular bone also facilitates osteoconduction, improving integration with host tissue and reducing healing time in critical-sized defects.

#### 4.3.2. Targeted Delivery and Bone-Specific Homing

A major translational goal is the development of delivery systems capable of targeting bone tissue with high specificity. One strategy under investigation involves decorating the EV membrane with bisphosphonate functionalisation (e.g., alendronate) to enhance hydroxyapatite binding and bone retention, thereby increasing localisation within mineralised compartments. [76,77,78]. Another emerging concept is the exploitation of physiological chemokine axes such as CXCL12/CXCR4, which naturally direct cell trafficking toward bone marrow niches [79,80]. Engineering EVs or biomaterials to engage these pathways could substantially improve localisation and mitigate off-target biodistribution.

Smart biomaterials capable of releasing EVs in response to pathological cues—such as acidic pH or elevated protease activity—offer additional specificity [81]. By restricting EV release to environments undergoing active inflammation or remodelling, these systems enhance therapeutic precision and minimise systemic exposure. Together, targeted homing and responsive delivery systems provide crucial technological advancements for increasing the efficacy and safety of EV-based interventions.

### 4.4. Manufacturing, Quality Control, and Regulatory Barriers

The transition from experimental systems to clinical-grade products requires overcoming significant manufacturing and regulatory hurdles. EV composition varies widely depending on MSC donor characteristics, culture conditions, passage number, and isolation method, resulting in considerable batch-to-batch variability [82]. Such variability poses challenges for defining potency assays and release criteria that reliably predict therapeutic performance. Beyond purely technical sources of variability, accumulating evidence demonstrates that the biological source of MSCs and donor-related factors critically shape the functional properties of the secretome and extracellular vesicles. MSCs derived from different tissues, including bone marrow, adipose tissue, and umbilical cord, exhibit distinct immunomodulatory and osteogenic profiles, reflecting intrinsic differences in developmental origin, baseline inflammatory sensitivity, and secretory programs. In addition, donor age, systemic inflammatory status, metabolic conditions, and underlying disease profoundly influence MSC behaviour, altering both the composition and biological activity of their secretome and EV cargo. Ageing and chronic inflammatory environments have been shown to impair MSC immunoregulatory capacity, skew EV-associated signalling toward reduced osteogenic potency, and in some contexts promote maladaptive or pro-fibrotic responses. These observations highlight that the MSCs-derived secretome and EVs should not be considered biologically uniform products, but rather context-dependent entities whose regenerative efficacy is shaped by source- and donor-specific variables. From a translational perspective, this biological heterogeneity reinforces the need for rigorous donor selection, functional characterisation, and potency assays that integrate both molecular cargo and biological activity, alongside manufacturing standardisation, to ensure predictable and safe clinical outcomes [18,59,60,64,82]. The development of standardised EV production pipelines that preserve vesicle integrity, biological function, and reproducibility is therefore essential.

Scaling EV production to clinically relevant volumes remains a key barrier. Ultracentrifugation, though common in laboratory settings, is difficult to translate to GMP-scale manufacturing. Emerging alternatives such as tangential flow filtration, size-exclusion chromatography, and high-density bioreactors show promise but require further validation to ensure that vesicle cargo and functionality remain intact. Moreover, regulatory agencies increasingly demand comprehensive data on biodistribution, pharmacokinetics, chronic toxicity, immune compatibility, and potential oncogenicity. Despite the favourable safety profile of EVs, rigorous long-term studies will be necessary to meet regulatory standards and support first-in-human trials.

### 4.5. Mechanistic Gaps and Future Biological Opportunities

Although significant progress has been made, key mechanistic questions remain unresolved. The bone microenvironment is a highly specialised ecosystem composed of osteoblasts, osteoclasts, osteocytes, endothelial cells, immune cells, and skeletal progenitors. Each cellular population may interpret EV-derived signals differently depending on its differentiation state, metabolic profile, and exposure to inflammatory cues [1,83]. Understanding how EV cargo is taken up, processed, and integrated into cellular decision-making remains an essential research frontier.

The emerging field of immunometabolism adds an additional layer of complexity to these interactions by revealing that immune cell function is tightly coupled to cellular metabolic state. Distinct metabolic programmes in macrophages, characterised by a predominantly glycolytic profile in M1 cells and an oxidative, mitochondrial metabolism in M2 cells, actively regulate inflammatory resolution and tissue repair rather than merely reflecting activation status. Early evidence indicates that the MSC secretome and EVs may modulate these metabolic states, thereby shifting immune responses toward a pro-regenerative phenotype [84]. However, the molecular mechanisms governing these effects remain poorly understood.

Recent advances in single-cell RNA sequencing and spatial transcriptomics provide powerful tools to dissect tissue-specific cellular niches and to begin resolving how distinct cell populations respond to EV-based interventions in vivo. These technologies enable precise mapping of target cell populations, reconstruction of signalling networks activated by EV uptake, and identification of biomarkers predictive of treatment response [85]. Integrating these tools into future studies will be essential for refining therapeutic strategies and improving translational predictability.

## 5. Conclusions and Future Perspectives

Over the past two decades, osteoimmunology has fundamentally reshaped our understanding of bone biology, revealing that skeletal homeostasis and regeneration are inseparable from immune regulation. Rather than being a passive structural tissue, bone is now recognised as a dynamic organ whose integrity depends on finely tuned interactions among stromal cells, immune populations, and vascular components. Within this framework, mesenchymal stem cells emerge as central coordinators of bone–immune communication, integrating inflammatory cues with regenerative responses.

Accumulating evidence indicates that the contribution of MSCs to bone repair extends far beyond their capacity for lineage differentiation. Instead, MSCs act primarily through paracrine mechanisms, deploying a complex secretome that modulates immune activation, supports osteogenic differentiation, and promotes angiogenesis. Soluble mediators and extracellular vesicles released by MSCs operate in concert to resolve inflammation, reprogramme immune cell phenotypes, and create a microenvironment permissive for tissue regeneration. This integrated mode of action explains why MSC-based interventions can exert broad and durable effects even in the absence of long-term cell engraftment.

Extracellular vesicles have emerged as particularly powerful mediators of these effects. By transferring proteins, lipids, and regulatory RNAs to specific target cells, MSCs-derived EVs enable precise and context-dependent modulation of osteoblasts, osteoclasts, macrophages, and lymphocytes. Importantly, EV cargo is not static, but reflects the physiological state of the parent MSCs, allowing environmental cues such as inflammation, hypoxia, or mechanical stimulation to be translated into tailored biological signals. This adaptability positions EVs as versatile regulators of osteoimmune homeostasis and as attractive candidates for therapeutic development.

The growing interest in cell-free strategies based on the MSC secretome and EVs reflects both biological insight and practical considerations. Compared with cell transplantation, acellular approaches offer advantages in terms of safety, standardisation, storage, and scalability. Preclinical studies consistently demonstrate that MSCs-derived EVs can enhance fracture healing, attenuate inflammation-driven bone loss, and promote vascularised bone regeneration. These findings support the notion that recreating the paracrine functions of MSCs may be sufficient to trigger effective repair in a wide range of skeletal pathologies.

Despite this progress, significant challenges remain before these strategies can be translated into routine clinical practice. The heterogeneity of EV preparations, which is influenced by MSC source, culture conditions, and isolation methods, complicates reproducibility and regulatory approval. In parallel, key mechanistic questions remain unresolved, particularly regarding how different cell populations within the bone microenvironment interpret EV-derived signals and how these responses are shaped by metabolic and inflammatory states. Addressing these gaps will require integrative approaches that combine functional assays with emerging technologies such as single-cell and spatial transcriptomics.

Looking ahead, the convergence of osteoimmunology, extracellular vesicle biology, and biomaterials science offers exciting opportunities. Advances in EV engineering, targeted delivery systems, and controlled release platforms are likely to improve therapeutic precision and efficacy. At the same time, a deeper understanding of immune-stromal interactions will enable more rational design of regenerative strategies tailored to specific disease contexts, such as osteoporosis, inflammatory arthritis, or impaired fracture healing.

In conclusion, MSCs and their secretome (particularly EVs) represent a unifying biological axis linking immune regulation and bone regeneration. By harnessing these mechanisms, future therapies may move beyond symptomatic treatment toward restoring the physiological balance required for long-term skeletal health. Continued interdisciplinary research will be essential to translate these insights into safe, effective, and clinically applicable regenerative solutions.

## Data Availability

No new data were created or analysed in this study. Data sharing is not applicable to this article.

## Abbreviations

AKT	Protein kinase B
ALP	Alkaline phosphatase
BMP	Bone morphogenetic protein
CXCL	C-X-C motif chemokine ligand
CXCR	C-X-C motif chemokine receptor
EVs	Extracellular vesicles
GMP	Good Manufacturing Practice
HGF	Hepatocyte growth factor
HIF-1α	Hypoxia-inducible factor 1 alpha
IDO	Indoleamine 2,3-dioxygenase
IFN-γ	Interferon gamma
IL	Interleukin
JNK	c-Jun N-terminal kinase
MMP	Matrix metalloproteinase
MSC	Mesenchymal stem/stromal cell
NF-κB	Nuclear factor kappa B
NK	Natural killer
OPG	Osteoprotegerin
PGE2	Prostaglandin E2
PI3K	Phosphoinositide 3-kinase
PTEN	Phosphatase and tensin homolog
RANK	Receptor activator of nuclear factor κB
RANKL	Receptor activator of nuclear factor κB ligand
RUNX2	Runt-related transcription factor 2
STAT3	Signal transducer and activator of transcription 3
TGF-β	Transforming growth factor beta
Th	T helper
TLR	Toll-like receptor
TNF-α	Tumour necrosis factor alpha
Treg	Regulatory T cell
VEGF	Vascular endothelial growth factor
